# Supplementary material for: Incidence of Air Leaks in Critically Ill Patients with Acute Hypoxemic Respiratory Failure Due to COVID-19
Source: Diagnostics (Basel). 2023 Mar 17;13(6):1156. doi: 10.3390/diagnostics13061156 (PMC10046975; doi:10.3390/diagnostics13061156)
Supplement: Supplementary file 1 [file diagnostics-13-01156-s001.zip › diagnostics-2276076-supplementary.pdf]

diagnostics

MDPI

*Article*

# Incidence of Air Leaks in Critically Ill Patients with Acute Hypoxemic Respiratory Failure Due to COVID-19

**Robin L. Goossen [1,*,†], Mariëlle Verboom [1,†], Mariëlle Blacha [1], Illaa Smesseim [2], Ludo F. M. Beenen [3], David M. P. van Meenen [1,4], Frederique Paulus [1,5] and Marcus J. Schultz [1,6,7,*] and on behalf of the PRoVENT–COVID and PRoAcT–COVID Investigators [‡]**

1 Department of Intensive Care, Amsterdam University Medical Centers, Location 'AMC', 1105 AZ Amsterdam, The Netherlands
2 Department of Thoracic Oncology, Antoni van Leeuwenhoek Ziekenhuis, 1066 CX Amsterdam, The Netherlands
3 Department of Radiology, Amsterdam University Medical Centers, Location 'AMC', 1105 AZ Amsterdam, The Netherlands
4 Department of Anesthesiology, Amsterdam University Medical Centers, Location 'AMC', 1105 AZ Amsterdam, The Netherlands
5 ACHIEVE, Centre of Applied Research, Faculty of Health, Amsterdam University of Applied Sciences, 1091 GC Amsterdam, The Netherlands
6 Mahidol–Oxford Tropical Medicine Research Unit (MORU), Mahidol University, Bangkok 10400, Thailand
7 Nuffield Department of Medicine, University of Oxford, Oxford OX3 7BN, UK
* Correspondence: r.l.goossen@amsterdamumc.nl (R.L.G.); marcus.j.schultz@gmail.com (M.J.S.)
† These authors contributed equally to this work.
‡ A full list of the collaborative investigators is provided Acknowledgments.

Supplementary

**Table S1.** Amount of missing data in Table 1.

| | Patients with air leak (N = 13) | Patients without air leak (N = 143) |
|---|---|---|
| Age, years | 0 (0.0) | 0 (0.0) |
| Sex, male | 0 (0.0) | 0 (0.0) |
| BMI, kg/m$^2$ | 2 (15) | 24 (16.8) |
| SOFA score | 4 (30.8) | 32 (22.4) |
| Medical History | | |
| COPD | 0 (0.0) | 0 (0.0) |
| asthma | 0 (0.0) | 0 (0.0) |
| lung emphysema | 0 (0.0) | 0 (0.0) |
| interstitial lung disease | 0 (0.0) | 0 (0.0) |
| active smoker | 7 (53.8) | 66 (46.2) |
| history of smoking | 8 (61.5) | 69 (48.3) |
| previous pneumothorax | 0 (0.0) | 0 (0.0) |
| Home medication | | |
| Ace inhibitors | 1 (7.7) | 28 (19.6) |
| Angiotensin receptor blocker | 1 (7.7) | 28 (19.6) |
| Ventilatory Support | | |
| with invasive ventilation | 0 (0.0) | 0 (0.0) |
| $FiO_2$, % | 0 (0.0) | 3 (2.1) |
| $V_T$ mL/kg PBW | 2 (15.4) | 41 (28.7) |
| PEEP, cm $H_2O$ | 0 (0.0) | 15 (10.5) |
| Pmax, cm $H_2O$ | 0 (0.0) | 15 (10.5) |

| | **Patients with air leak (N = 13)** | **Patients without air leak (N = 143)** |
|---|---|---|
| $C_{RS}$, mL/cm$H_2O$ | 5 (38.5) | 52 (36.4) |
| with high flow nasal oxygen | 0 (0.0) | 0 (0.0) |
| $FiO_2$, % | N.A. | 0 (0.0) |
| flow, L $O_2$/min | N.A. | 0 (0.0) |
| Horowitz index | | |
| $PaO_2/FiO_2$ | 0 (0.0) | 2 (1.4) |
| $PaO_2/FiO_2 > 300$ mmHg | N.A. | N.A. |
| PaO2/$FiO_2$ 200–300 mmHg | N.A. | N.A. |
| $PaO_2/FiO_2$ 100–200 mmHg | N.A. | N.A. |
| $PaO_2/FiO_2 < 100$mmHg | N.A. | N.A. |

Data are N (%)

**Table S2.** Amount of missing data in Table 3.

| | **Patients with air leak (N = 13)** | **Patients without air leak (N = 143)** |
|---|---|---|
| Duration of respiratory support, days | 0 (0.0) | 0 (0.0) |
| ICU length of stay, days | 0 (0.0) | 0 (0.0) |
| ICU mortality | 0 (0.0) | 0 (0.0) |
| 28-day mortality | 0 (0.0) | 0 (0.0) |
| 90-day mortality | | |

Data are N (%)

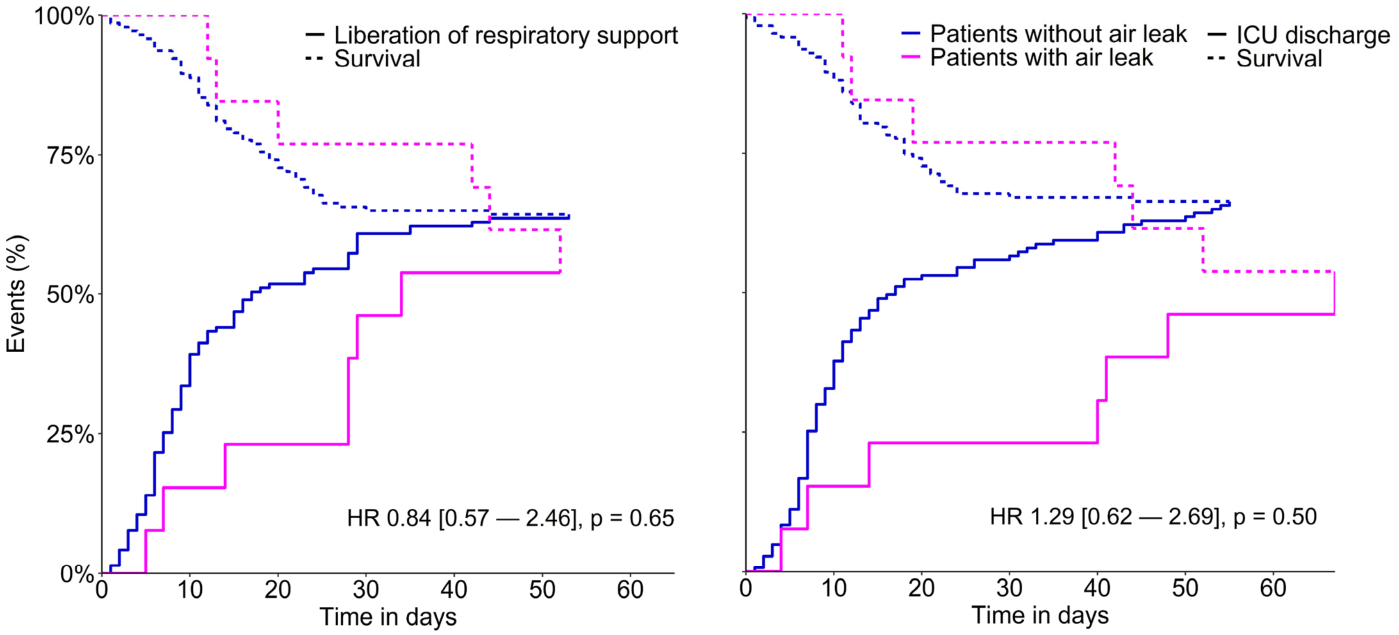


**Figure S1. Liberation of ventilation and ICU discharge.**
Cumulative incidence of liberation of respiratory support (left panel) and ICU discharge (right panel) with death as a competing risk, in patients with and without an air leak.
